# The Repertoire of Archaea Cultivated from Severe Periodontitis

**DOI:** 10.1371/journal.pone.0121565

**Published:** 2015-04-01

**Authors:** Hong T. T. Huynh, Marion Pignoly, Vanessa D. Nkamga, Michel Drancourt, Gérard Aboudharam

**Affiliations:** 1 Faculty of Dentistry, Aix Marseille University, 27, Boulevard Jean Moulin-Cedex 5, Marseille, France; 2 Unité de Recherche sur les Maladies Infectieuses et Tropicales Emergentes (URMITE), UMR CNRS 7278, IRD 198, INSERM 1095. Faculté de Médecine, 27, Boulevard Jean Moulin-Cedex 5, Marseille, France; Medical University Graz, AUSTRIA

## Abstract

In previous studies, the abundance and diversity of methanogenic archaea in the dental microbiota have been analysed by the detection of specific DNA sequences by PCR-based investigations and metagenomic studies. Few data issued regarding methanogens actually living in dental plaque. We collected dental plaque specimens in 15 control individuals and 65 periodontitis patients. Dental plaque specimens were cultured in an anoxic liquid medium for methanogens in the presence of negative control tubes. Dental plaque methanogens were cultured from 1/15 (6.67%) control and 36/65 (55.38%) periodontitis patient samples (p<0.001). The cultures yielded *Methanobrevibacter oralis* in one control and thirty-one patients, *Methanobrevibacter smithii* in two patients and a potential new species named *Methanobrevibacter* sp. strain N13 in three patients with severe periodontitis. Our observations of living methanogens, strengthen previous observations made on DNA-based studies regarding the role of methanogens, in periodontitis.

## Introduction

In previous studies, the abundance and diversity of methanogenic archaea in the dental microbiota have been analysed by the detection of specific DNA sequences by PCR-based investigations and metagenomic studies [1]. These analyses have revealed that the dominant methanogenic archaea in the oral cavity was *Methanobrevibacter oralis* [1–6]. A few isolates have been tentatively identified as *Methanobrevibacter smithii* and *Methanosphaera stadtmanae* using immunological methods, yet these identifications have not been confirmed by more conventional methods [7]. The presence of *M*. *smithii* in periodontal pockets has been further confirmed by molecular methods [8]. PCR-sequencing-based studies afterwards have detected *Methanosarcina mazeii* in both periodontitis patients and healthy subjects [3], *Thermoplasmata* spp. in periodontal pockets, but not in healthy subjects [5,6] and *Methanobacterium curvum/congolense* in periodontitis and peri-implantitis patients, also in pockets and healthy sites [2,3]. The sequences indicative of original phylotypes corresponding to yet uncultured methanogens have also been found [6,8–10]. However, such molecular approaches do not ensure the culturability of methanogens, and, currently, *M*. *oralis* is the sole methanogen isolated and firmly identified from dental microbiota [11]. Additional organisms have been implicated in periodontitis [1], and the severity of periodontitis has been significantly linked to the load of *M*. *oralis* using a specific real-time PCR assay [12]. Whether other methanogenic archaea reside in diseased dental pockets remains unknown.

In an effort to broaden the knowledge of the repertoire of methanogenic archaea actually living in dental microbiota, we utilized a culture-based approach and recovered for the first time a previously uncultured methanogen [8–10] in addition to *M*. *smithii* and *M*. *oralis*.

## Patients and Methods

Each participant provided a written informed consent to participate in this study. The consent form and procedure, as well as this research protocol, were approved by the ethics committee of the Institute "Institut Fédératif de Recherche 48", Marseille, France with number of agreement 12–008 on 6 February 2012. From October 2013 to March 2014, 15 healthy, control individuals and 65 periodontitis patients were prospectively enrolled in the Department of Odontology, Timone Hospital, Marseille. All the individuals were interviewed for medical history, dental history and smoking habits and had an intraoral examination for bleeding on probing, probing depth, plaque index, calculus index, presence of recession, mobility of teeth and tooth loss. The total score was determined according to a previously reported scale [13]. Patients and control individuals could be grouped into group A (low risk group) with a total score ranging from 0 to 16, group B (moderate risk group) from 17 to 32 and group C (high risk group) from 33 to 40. All individuals informed that they had not been exposed to antibiotics before plaque samples were collected. Subgingival dental plaque samples were collected from all periodontal pockets of each individual with sterile Gracey curettes 1/2 (Hu-Friedy, Rotterdam, Netherlands) and placed into Hungate tubes containing 5 mL of the SAB anoxic medium for methanogens composed of NiCl_2_.6H_2_0, 0.07 mg/L; FeSO_4_.7H_2_O, 0.2 mg/L; MgSO_4_.7H_2_O, 0.1 g/L; K_2_HPO_4_, 0.5 g/L; KH_2_PO_4_, 0.5 g/L; KCl 0.05 g/L; CaCl_2_, 0.05 g/L; NaCl, 1.5 g/L; NH_4_Cl, 1 g/L; NaAcetate, 1 g/L; yeast extract, 1 g/L; biotrypcase, 1 g/L; L-cysteine.HCl, 0.5 g/L; trace elements Widdel, 1 mL/L; resazurin, 1 mL/L; NaHCO_3_, 10%; Na_2_S, 2%; vancomycin, 100 mg/L, pH 7.5 with 10 M KOH [14]. The tubes inoculated with dental plaque and four negative control tubes containing non-inoculated medium were washed by a flux of nitrogen and were directly incubated at 37°C with agitation under a mixture of 80% H_2_ + 20% CO_2_ at 2-bar pressure. The growth of methanogens was monitored by measuring methane in the tubes using gas chromatography (Clarus 500, Perkin Elmer, Courtaboeuf, France).

All cultures were then screened for *M*. *oralis* using a specific real-time PCR assay targeting the heat-shock protein *cnp*60 gene of *M*. *oralis* as previously described [12]. Distilled water was used as negative control. A Ct value of >32 was considered as negative. All *M*. *oralis*-negative tubes were then screened for the presence of other methanogens using previously described PCR-sequencing of the partial methyl-coenzyme M redutase (*mcr*A) gene [15] and the 16S rRNA gene [16]. The sequences were analyzed with the ChromasPro program, version 1.5, and similarity values were determined by BLAST program in the online analysis platform from NCBI production was detected (blast.ncbi.nlm.nih.gov). The *mcr*A and 16S rRNA gene sequence-based phylogenetic trees were reconstructed using the neighbour-joining and maximum-likelihood tools implemented in the MEGA 5.2 software package [17].

The isolation of any cultured methanogenic archaea was performed according to the Hungate roll-tube method [18]. A 0.5 mL-volume of each Hungate tube in which methane was transferred into a tube of 5 mL melted agar medium in the water bath of 50°C and this tube was inverted to mix the inoculum. A 0.5 mL-volume of agar medium was transferred from the first tube to the second tube and inverted to mix the inoculum. A serial dilution through eight tubes of agar medium was generated likewise. The roll tubes were formed by rotating the tubes of agar medium under cold. These roll tubes were incubated using a gas mixture of H_2_/CO_2_ (80:20, v/v; at 2-bar pressure) at 37°C in an upright position. Four non-inoculated tubes followed the same procedure. Identification of colonies was done by 16S rRNA gene PCR-sequencing.

The t-test was used to compare the sex ratio and age range of patient group and control group, the Mann-Whitney test to compare the total score of the groups. The χ^2^ test was used to test differences in the prevalence rates of methanogenic archaea between two groups. Difference of the severity of three periodontitis patients who have the newly isolated archaea and that of the patients who have *M*. *oralis* was also evaluated.

## Results and Discussion

The groups had different sex ratio (male / female) (1.5 and 0.91, respectively) and age range (23–61, mean 38.3 years; 25–79, mean 53.8 years, respectively) (p<0.001, *t*-test). The total score varied from 8 to 26 in the patient group and from 1 to 14 in the control group (p<0.01, Mann-Whitney test). Group A (low risk) comprised 42 individuals (52.5%), including 15 (35.71%) controls and 27 (64.29%) patients; group B (moderate risk) included 38 patients (47.5%), and there was no individual in group C (high risk).

**Fig 1:**
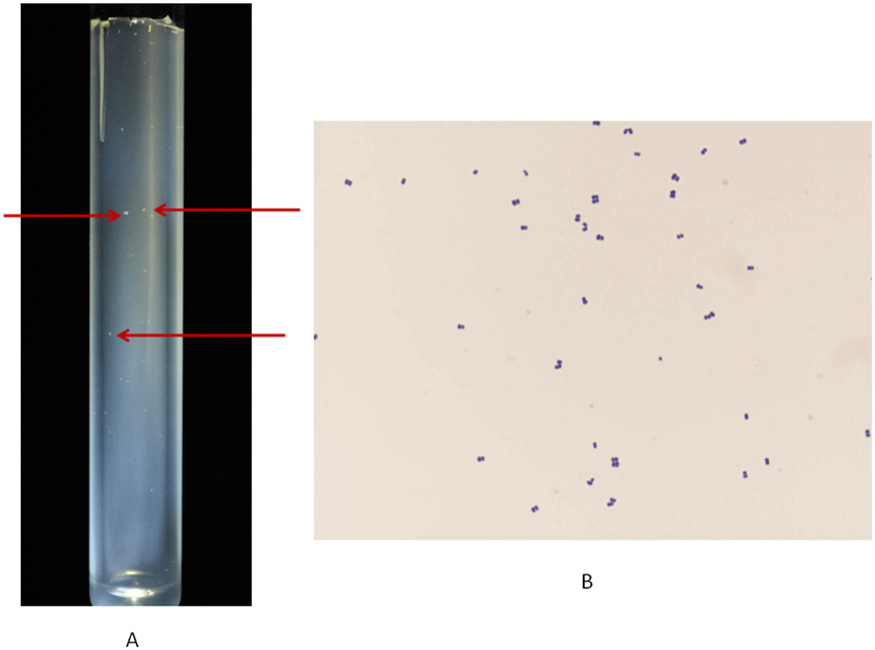
*Methanobrevibacter* sp. strain N13. (A) Colonies on SAB medium (arrows). (B) Gram-staining.

**Fig 2:**
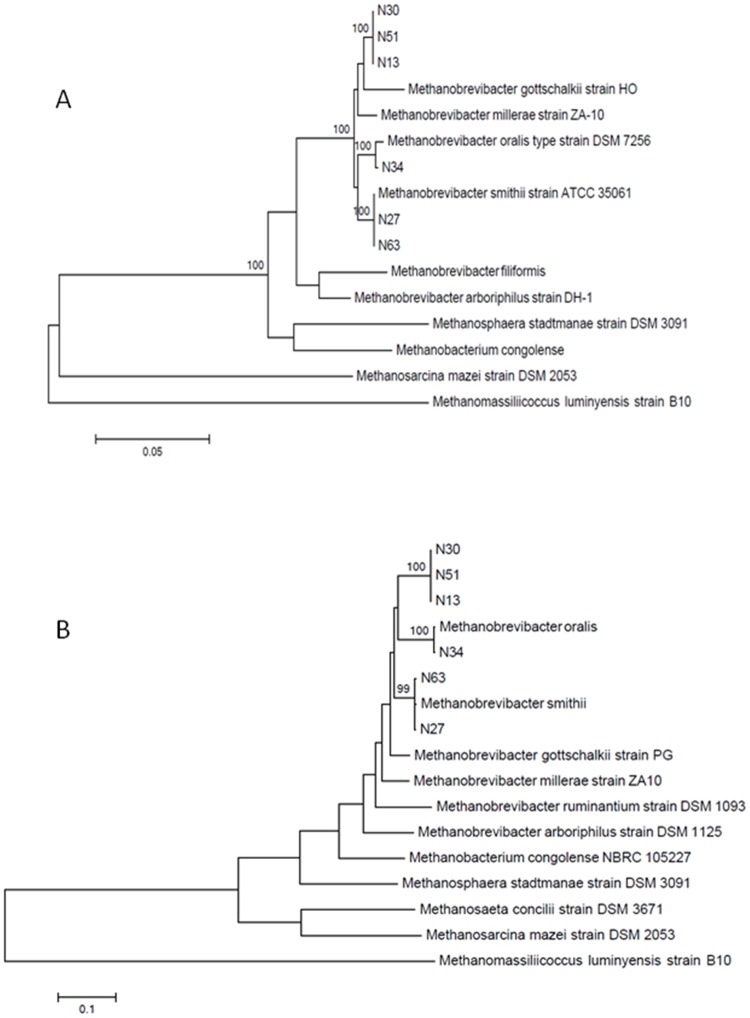
A, 16S rRNA gene sequence-based phylogenetic tree. B, *Mcr*A gene sequence-based phylogenetic tree. Phylogenetic position of *Methanobrevibacter* sp. strain N13 cultured from the dental plaque of three patients with severe periodontitis (N13, N30, N51). Bootrap values ≥ 95% are indicated at nodes. The scale bar represents phylogenetic distance.

Methane was not detected in any of the control tubes but was detected in 1/15 (6.67%) tube from the control group and in 36/65 (55.38%) tubes from the patient group (p<0.001). Real-time PCR was negative in tubes that did not produce methane and positive in the methane-producing tube in the control group and in 31/36 (86.11%) methane-producing tubes in the patient group (p<0.001). Combined together, these data indicate that the prevalence of living methanogens is significantly higher in periodontitis patients than in the control group. This is a new set of data, as previous studies have been all based on the detection of specific DNA sequences, not cultured archaea. Concerning five tubes which produced methane but remained negative in *M*. *oralis* real-time PCR, *M*. *smithii* was identified in tubes N27 and N63, with 100% 16S rRNA gene sequence similarity with the reference (NR_074235.1) and 99% *mcr*A sequence similarity with the reference (CP000678.1). Moreover, a previously uncultured *Methanobrevibacter* sp. organism reported as "phylotype 3" was cultured in tubes N13, N30 and N51, with 100% 16S rRNA gene sequence similarity (AJ001711; FJ755684) [8,9] and 99% *mcr*A sequence similarity (FJ755685; EU294498) [9,10]. This microorganism exhibited 98% 16S rRNA gene sequence similarity with the nearest named species *Methanobrevibacter gottschalkii* (gb|U55239.1|MSU55239) with 20/1,258 bases of difference and *Methanobrevibacter millerae* (NR_042785.1) with 21/1,257 bases of difference. Its *mcr*A sequence showed 90% similarity with *M*. *gottschalkii* (EU919431.1) with 41/425 bases of difference and 89% similarity with *M*. *smithii* (DQ251046.1) with 45/425 bases of difference. The negative controls remained negative in all the PCR-based experiments. This methanogen was further referred as *Methanobrevibacter* sp. strain N13. Microscopic examination of colonies indicated a Gram-positive organism forming pairs, tetrads and short chains, (Fig 1). The 16S rRNA and *mcr*A gene sequence-based phylogenetic trees confirmed the BLAST data and the uniqueness of *Methanobrevibacter* sp. strain N13 (Fig 2). All sequences reported here have been deposited in GenBank (LK054623-LK054628 for *mcr*A gene sequences; LK054632-LK054637 for 16S rRNA gene sequences). *Methanobrevibacter* sp. strain N13 was deposited in our publicly available collection “Collection de Souches de l’Unité des Rickettsies” (CSUR P1375).

In this study, the prevalence of *M*. *oralis* was significantly higher in the patients (31/65; 47.69%) than in the control group (1/15; 6.67%) (p<0.01). The prevalence of any living archaeon was significantly higher (25/38; 65.79%) in the moderate risk group B than in group A (12/42; 28.57%) (p<0.01) (Table 1). In particular, the patients hosting *M*. *smithii* or *Methanobrevibacter* sp. strain N13 organisms had the highest scores in our study, including scores of 22 and 25 (*M*. *smithii*) and 15, 24 and 26 (*Methanobrevibacter* sp. strain N13). One *Methanobrevibacter* sp. strain N13-positive patient had remarkably severe chronic periodontitis, with deep probing up to 15 mm in 10 sites and up to 10 mm in 30 sites, generalized bleeding on probing and 7 mobile teeth. Another *Methanobrevibacter* sp. strain N13-positive patient had severe chronic periodontitis, with 5–7 mm probing depth in 60% sites, general gingival recession and four mobile teeth. A third *Methanobrevibacter* sp. strain N13-positive patient had also typical chronic periodontitis, with 5–6 mm probing depth in 30% sites, general gingival recession and 11 lost posterior teeth. One patient positive for *M*. *smithii* had 5–7 mm probing depth in 30% sites, up to 10 mm probing depth in two sites and 13 lost anterior and posterior teeth.

**Table 1:** Periodontitis score and the archaea cultured using dental plaque collected from patients with periodontitis and controls.

Patient	Total score^a^	Archaea
N1	11	*M*. *oralis*
N2	10	*M*. *oralis*
N3	20	*M*. *oralis*
N4	14	*-*
N5	16	*M*. *oralis*
N6	17	*M*. *oralis*
N7	20	*-*
N8	14	*-*
N9	17	*M*. *oralis*
N10	22	*M*. *oralis*
N11	15	*-*
N12	16	*-*
N13	15	*Methanobrevibacter* sp. strain N13
N14	22	*M*. *oralis*
N15	16	*M*. *oralis*
N16	20	*M*. *oralis*
N17	17	*M*. *oralis*
N18	20	*M*. *oralis*
N19	11	*-*
N20	17	*M*. *oralis*
N21	19	*-*
N22	9	*-*
N23	15	*M*. *oralis*
N24	22	*M*. *oralis*
N25	16	*-*
N26	15	*M*. *oralis*
N27	22	*M*. *smithii*
N28	24	*-*
N29	26	*M*. *oralis*
N30	26	*Methanobrevibacter* sp. strain N13
N31	19	*M*. *oralis*
N32	8	*-*
N33	13	*M*. *oralis*
N34	24	*M*. *oralis*
N35	17	*-*
N36	19	*-*
N37	11	*M*. *oralis*
N38	17	*M*. *oralis*
N39	21	*M*. *oralis*
N40	18	*M*. *oralis*
N41	23	*M*. *oralis*
N42	21	*M*. *oralis*
N43	16	*-*
N44	18	*-*
N45	15	*-*
N46	14	*-*
N47	16	*M*. *oralis*
N48	8	*-*
N49	11	*-*
N50	14	*M*. *oralis*
N51	24	*Methanobrevibacter* sp. strain N13
N52	16	*-*
N53	21	*-*
N54	13	*-*
N55	9	*-*
N56	20	*-*
N57	17	*-*
N58	17	*M*. *oralis*
N59	17	*M*. *oralis*
N60	20	*M*. *oralis*
N61	21	*-*
N62	21	*-*
N63	25	*M*. *smithii*
N64	19	*-*
N65	18	*-*
**Control**		
C1	5	*-*
C2	4	*-*
C3	2	*-*
C4	1	*-*
C5	3	*-*
C6	5	*-*
C7	4	*-*
C8	5	*-*
C9	3	*-*
C10	1	*-*
C11	14	*-*
C12	5	*-*
C13	8	*M*. *oralis*
C14	11	*-*
C15	4	*-*

^a^ The score was determined according to Chandra RV [13].

After four-month incubation, colonies developed in roll tubes in 21 specimens, including nineteen *M*. *oralis* tubes, one *M*. *smithii* (N27) and one *Methanobrevibacter* sp. strain N13 (N30). Colonies in N27 and N30 were also identified with 16S rRNA PCR-sequencing as *M*. *smithii* and *Methanobrevibacter* sp. strain N13. No colony was observed in control roll tubes. *Methanobrevibacter* sp. strain N13 was deposited in our publicly available collection (CSUR P1375).

This culture-based study increases knowledge of the repertoire of methanogenic archaea living in dental microbiota, particularly in diseased dental pockets. We cultured *M*. *oralis* in 47.7% of periodontitis patients, a figure in the range of previously published data [1]. *M*. *oralis* was also cultured from one healthy individual (6.7%), in line with the previously reported 6% prevalence of *M*. *oralis* in healthy individuals using molecular detection [1]. Other PCR-based studies found a higher prevalence of *M*. *oralis* DNA in healthy individuals, illustrating that PCR-based studies may overestimate the actual prevalence of methanogens by detecting DNA from dead organisms. *M*. *smithii*, the dominant methanogenic archaeon in the human gut, was cultured for the first time from two periodontitis patients. This methanogen has been detected only by PCR-based approaches in previous studies [4,8]. Indeed, previous dental plaque isolates were tentatively identified by immunological methods; using DNA sequencing, we here confirm the identification of *M*. *smithii* isolates [7]. *M*. *smithii* has recently been shown to be able to induce human immune responses [19], which could be involved in the severity of periodontal disease. More surprisingly, *Methanobrevibacter* sp. train N13, a previously uncultured organism referred as "phylotype 3" [8,9] was cultured from three patients. This methanogen is close to *M*. *gottschalkii* which had been detected only in ruminants and isolated from enrichments of horse and pig feces [20], though none of the previous molecular studies, including advanced metagenomic studies, detected *M*. *gottschalkii* archaea in the human oral cavity [1]. Our results confirm the results obtained by PCR-based studies by proving the viability of *Methanobrevibacter* sp. strain N13 archaeon in addition to *M*. *smithii* and *M*. *oralis* in oral cavity. This observation therefore illustrates the complementarity of culture-based and culture-independent approaches to broaden knowledge of the spectrum of archaeon repertoires in the oral cavity.

## Conclusions

Establishing the repertoire of methanogens living in the dental microbiota is of interest, as methanogens [21] and *M*. *oralis* in particular [1] have been implicated in periodontitis. Accordingly, the prevalence of methanogens was significantly higher in periodontitis patients than in controls. In particular, the *Methanobrevibacter* sp. strain N13 organism may not be a mere bypasser, as it has been cultured independently from three independent patients but not from healthy individuals. Our observations of living methanogens, strengthen previous observations made on DNA-based studies regarding the role of methanogens, in periodontitis.
